# Gut microbiota affects obesity susceptibility in mice through gut metabolites

**DOI:** 10.3389/fmicb.2024.1343511

**Published:** 2024-02-21

**Authors:** Yuhang Wen, Yadan Luo, Hao Qiu, Baoting Chen, Jingrong Huang, Shuya Lv, Yan Wang, Jiabi Li, Lingling Tao, Bailin Yang, Ke Li, Lvqin He, Manli He, Qian Yang, Zehui Yu, Wudian Xiao, Mingde Zhao, Xiaoxia Zou, Ruilin Lu, Congwei Gu

**Affiliations:** ^1^Laboratory Animal Centre, Southwest Medical University, Luzhou, China; ^2^Model Animal and Human Disease Research of Luzhou Key Laboratory, Luzhou, China; ^3^Suining First People's Hospital, Suining, China

**Keywords:** susceptibility to obesity, gut microbiota, untargeted metabolomics, high-fat diet, obesity-prone, obesity-resistant

## Abstract

**Introduction:**

It is well-known that different populations and animals, even experimental animals with the same rearing conditions, differ in their susceptibility to obesity. The disparity in gut microbiota could potentially account for the variation in susceptibility to obesity. However, the precise impact of gut microbiota on gut metabolites and its subsequent influence on susceptibility to obesity remains uncertain.

**Methods:**

In this study, we established obesity-prone (OP) and obesity-resistant (OR) mouse models by High Fat Diet (HFD). Fecal contents of cecum were examined using 16S rDNA sequencing and untargeted metabolomics. Correlation analysis and MIMOSA2 analysis were used to explore the association between gut microbiota and intestinal metabolites.

**Results:**

After a HFD, gut microbiota and gut metabolic profiles were significantly different between OP and OR mice. Gut microbiota after a HFD may lead to changes in eicosapentaenoic acid (EPA), docosahexaenoic acid (DHA), a variety of branched fatty acid esters of hydroxy fatty acids (FAHFAs) and a variety of phospholipids to promote obesity. The bacteria *g_Akkermansia* (Greengene ID: 175696) may contribute to the difference in obesity susceptibility through the synthesis of glycerophosphoryl diester phosphodiesterase (glpQ) to promote choline production and the synthesis of valyl-tRNA synthetase (VARS) which promotes L-Valine degradation. In addition, gut microbiota may affect obesity and obesity susceptibility through histidine metabolism, linoleic acid metabolism and protein digestion and absorption pathways.

## 1 Introduction

Obesity is one of the main health problems worldwide, with more than 1 billion obese people reported globally (United Nations, 2022). The obesity rate has been consistently increasing worldwide (Geng et al., 2022). The occurrence of obesity is influenced by various factors, such as genetics, dietary behavior, individual activity, and energy expenditure (Reddon et al., 2016; Bluher, 2019). Additionally, obesity is causally linked to diseases like diabetes, hypertension, and heart disease (Collaborators et al., 2017). It is urgent to address the health crisis of obesity. Studies have shown that individuals differ in their susceptibility to obesity, even if they have the same genetic background and dietary environment (Gu et al., 2019). Obesity sensitive animals induced by HFD are defined as the obesity-prone (OP) phenotype, while those that are not sensitive are defined as the obesity-resistant (OR) phenotype (Zhang et al., 2018).

The gut microbiome is considered to be the “second human genome” that controls human health and may affect the body's energy balance (Turnbaugh et al., 2006). Intestinal microbial imbalance can alter the function of gut barriers and gut associated lymphoid tissues (GALT), allowing bacterial components (such as LPS) to pass through the intestinal wall. This can induce the production of inflammatory cytokines and promote the development of insulin resistance. Additionally, it can also alter gastrointestinal peptide production and lipid metabolism, increase food intake, and contribute to body obesity (Gomes et al., 2018). At the same time, the susceptibility to obesity is also influenced by gut microbiota. Studies have demonstrated significant differences in the gut microbiota between OP mice and OR mice, with a decrease in the proportion of *Firmicutes* and *Bacteroides*, and an increase in the proportion of *Proteobacteria*. The enrichment of *Parasutterella* from the *Proteobacteria* phylum in OP mice is essential for the development of obesity (Gu et al., 2019). *C. Butyricum*, when enriched in the rat gut, can participate in energy metabolism and storage (Obanda et al., 2021). The decrease of *Bacteroidetes* and *Firmicutes* in the intestinal tract of mice leads to ineffective absorption of carbohydrates, which may result in obesity resistance (Li et al., 2014). Dysregulation of the BA signaling pathway mediated by intestinal flora also leads to obesity susceptibility (Wei et al., 2020).

Existing studies have explored the influence of gut microbiota on obesity susceptibility through metagenomic and serum metabolome methods (Gu et al., 2019; Wei et al., 2020). However, the effect of gut microbiota on obesity susceptibility through gut metabolites is inconclusive. In this study, we induced obesity-prone (OP) and obesity-resistant (OR) mouse models to investigate the differences in gut microbiota and metabolites in mice with different susceptibility to obesity. We used 16S rDNA sequencing and non-targeted metabolomics methods for this purpose. Additionally, we aimed to explore the mechanism by which gut microbiota affects obesity susceptibility through gut metabolites. The findings of this study will provide a scientific basis for improving obesity susceptibility by adjusting gut microbiota.

## 2 Materials and methods

### 2.1 Animal feeding and sample collection

Six-weeks-old specific pathogen-free male C57BL/6 mice (weighing 17–19 g) were purchased from Beijing Weitong Lihua Laboratory Animal Technology Co., LTD and housed at 22 ± 2°C and 50%-60% relative humidity in a specific pathogen-free facility maintained on a 12-h light/dark cycle in the Laboratory Animal Center of Southwest Medical University. After 1 week of acclimatization, 60 mice were randomly divided into two groups for a period of 10 weeks: the control (CK) group (*n* = 15) and the high-fat diet (HFD) group (*n* = 45). The CK mice were fed a normal diet, while the HFD mice were fed a high-fat diet. The normal diet comprised 65.08 kcal% carbohydrates, 23.07 kcal% proteins and 11.85 kcal% fats, while the HFD diet contained 20 kcal% carbohydrates, 20 kcal% proteins and 60 kcal% fats. The compositions of the normal and HFD diet are detailed in Supplementary Table 1. All experimental animals ate and drank freely. By week 9, mice on a high-fat diet that were 1.2 times heavier than the mean weight of CK mice were defined as obesity-prone (OP, *n* = 14). Those weighing <1.1 times the mean weight of CK mice were defined as obesity-resistant (OR, *n* = 14), and the remaining mice were eliminated. Four mice in each group were randomly selected for glucose tolerance and insulin tolerance tests, while the remaining mice were kept on the original diet for another week. At the end of the prescribed feeding period, the remaining mice were fasted overnight, anesthetized by intraperitoneal injection of 1% pentobarbital sodium (50 mg/kg body weight) and then sacrificed by cervical dislocation. Samples of liver and perirenal and epididymal fat were collected and weighed immediately. The cecum contents below the ileocecal valve were collected and frozen at −80°C for 16S microbiome and non-targeted metabolome detection.

### 2.2 Glucose tolerance test

At week 9, randomly selected mice were fasted for 12 h. Blood samples were collected from the tail vein and blood glucose concentration was measured using a glucometer (Roche, ACCU-CHEK). Additionally, a 10% glucose solution (2 g/kg) was injected intraperitoneally. Blood glucose levels were monitored at 15, 30, 60, 90, and 120 min after administration. At the end of the test, the mice were kept on their original diet for another week.

### 2.3 Insulin tolerance test

At week 10, randomly selected mice were fasted for 4 h, and blood glucose concentrations were measured using the same method described above. This was followed by an intraperitoneal injection of 0.0075 IU/mL insulin solution at a dosage of 0.75 IU/kg. Blood glucose levels were monitored at 30, 60, 90, and 120 min after the administration.

### 2.4 Biochemical analysis of serum

Blood samples were collected in the morning and centrifuged at 4°C at 3,500 r/min for 10 min. Serum was collected for testing triglyceride (TG), total cholesterol (TC), low density lipoprotein (LDL) and high density lipoprotein (HDL) by a fully automatic veterinary biochemical analyzer (Jiangxi Tekang Technology Co., Ltd., TC220).

### 2.5 Liver histopathology

Freshly isolated liver was soaked and fixed in 4% paraformaldehyde. After rinsing with running water, the tissues were dehydrated, embedded in paraffin, sectioned at 4 μm and stained with hematoxylin and eosin. Histopathological characteristics of each sample were assessed under the microscope and three photographs were taken under a light microscope with ×100 and ×400 magnification. The NAFLD activity scoring (NAS) was performed according to the Kleiner score system (Kleiner et al., 2005) in accordance with the National Institutes of NASH Clinical Research Network Pathology Working Group guidelines. Freshly isolated livers were embedded in optimal cutting temperature compound (OCT) and cryogenically preserved at −80°C. Subsequently, the tissues were sectioned at 4μm and stained with Oil red O (RUIBIO, Y07512). Histopathological characteristics of each sample were assessed under the microscope and three photographs were taken under a light microscope with ×400 magnification. Image Pro Plus was used to measure and analyze the lipid droplet area in liver tissue.

### 2.6 Fat histopathology

Freshly isolated adipose tissue around the epididymis was soaked and fixed in 4% paraformaldehyde. After rinsing with running water, the tissues were dehydrated, embedded in paraffin, sectioned at 4 μm and stained with hematoxylin and eosin. Histopathological characteristics of each sample were assessed under the microscope and three photographs were taken under a light microscope with × 400 magnification. Image Pro Plus was used to measure and analyze the adipocyte area.

### 2.7 16S rDNA gene sequencing

Genomic DNA extraction kit (D3141, Guangzhou Meiji Biological Co., LTD., China) was used to extract genomic DNA from cecum contents. The V3-V4 region of 16S rDNA (341F, CCTACGGGRBGCASCAG; 806R: GGACTACNNGGGTATCTAAT) was amplified using a specific primer with Barcode sequence. The amplified products were purified, quantified by ABI StepOnePlus RealTime PCR System (Life technologies, USA) and sequenced by computer. According to the sequences of different samples of specific markers, through the use of FLASH (V1.2.11, http://ccb.jhu.Edu/software/FLASH/), joining together the sequence for raw reads. DADA2 (V1.14.1, https://benjjneb.github.io/dada2/) software was used to filter and control raw reads. Amplicon Sequence Variants of single base accuracy were clustered, which was equivalent to OTU of 100% similarity clustering. The SILVA database was used to compare taxonomic information, and microbial composition analysis was performed at the phylum, class, order, family, genus and species levels. QIIME (V1.9.1, http://qiime.org/) software was used for Alpha diversity analysis and R software was used for Alpha diversity index and Beta diversity analysis. LEfSe was used to analyze the differential abundance of the microbes. Results with LDA scores >4 were retained by default in this experiment to determine the genus of marker bacteria in each group. PICRUSt2 was used to predict the metabolic pathways of the gut microbiota.

### 2.8 Untargeted metabolomics

A 100 mg tissue sample grounded in liquid nitrogen was added to 500 μL of 80% methanol solution for Vortex oscillation, and then placed in ice bath for 5 min and centrifuged at 15,000 g at 4°C for 20 min. The supernatant was collected and diluted to 53% methanol content, centrifuged again for 20 min, and then the supernatant was collected again and injected into LC-MS for analysis. Equal volume samples were taken from each experimental sample and mixed as quality control (QC) samples. The Vanquish UHPLC system (ThermoFisher, Germany) combined with the Orbitrap Q ExactiveTM HF-X mass spectrometer (Thermo Fisher, Germany) was used for UHPLC-MS/MS analysis. The samples were injected into a Hypesil Gold column (100×2.1 mm, 1.9μm) with a linear gradient of 17 min and a flow rate of 0.2 mL/min. The positive ion mode mobile phase A was 0.1% formic acid and the mobile phase B was methanol. Negative ion mode mobile phase A was 5 mM ammonium acetate (pH 9.0) and mobile phase B was methanol. Solvent gradient setting: 2% B, 1.5 min; 2–100% B, 12.0 min; 100% B, 14.0 min; 100–2% B, 14.1 min; and 2% B, 17 min. Q ExactiveTM HF-X working conditions of the mass spectrometer: spray voltage of 3.2 kV, capillary temperature of 320°C, sheath gas flow rate of 40 arb and aux gas flow rate of 10 arb. The raw data file generated by UHPLC-MS/MS was peak aligned, peak picked and quantified for each metabolite using Compound Discoverer 3.1 (CD3.1, Thermo Fisher). The main parameters were set as follows: retention time tolerance, 0.2 min; actual mass tolerance, 5ppm; signal intensity tolerance, 30%; signal/noise ratio, 3; and minimum intensity, 100,000. After that, a blank sample was used to remove the ionic background and the peak intensity was normalized to the total spectral intensity. Based on additive ions, molecular ion peaks and fragment ions, normalized data was used to predict molecular formula. It was compared with mzCloud (https://www.mzcloud.org/), mzVault and Masslist databases to obtain accurate qualitative and relative quantitative results.

### 2.9 Statistical analysis

In this study, GraphPad Prism 9.0.0 was used to calculate the area under the curve and SPSS 24.0 statistical software was used for statistical analysis. The *Shapiro-Wilk test* was employed to assess the normality of the data, while the *Levene test* was utilized to examine the homogeneity of variance. The data that adhered to a normal distribution and exhibited homogeneity of variance were subjected to single-factor ANOVA. Conversely, the data that adhered to a normal distribution but displayed heterogeneity of variance were analyzed using the *Welch test*. Lastly, the data that did not conform to a normal distribution were analyzed using the *Kruskal-Wallis rank sum test*. All values were expressed as mean ± SD. Spearman statistical method was used to analyze the correlation coefficient between significantly different metabolites and marker microorganisms in the test samples. In addition, R language (V2.15.3, http://www.R-project.org/) and Cytoscape software (V3.8.2, https://cytoscape.org/) were used for matrix heat mapping, hierarchical clustering and related network analysis. *P* < 0.05 indicates that there were significant differences in the experimental results.

MIMOSA2 analysis was used to further explore the relationship between intestinal flora and intestinal metabolites. MIMOSA2 analysis is a regression analysis that predicts microbial metabolic potential based on database and correlates the predicted metabolic potential with actual metabolomics data (Noecker et al., 2022). MIMOSA2 first compared all the ASV sequences in the 16S rDNA sequencing results with the data in KEGG, NCBI, EMBL-EBI, VMH and other databases, and constructed a network prediction model of the metabolic capacity of each microbial unit according to the sequence abundance. This metabolic network prediction model was then used to calculate the Metabolic Potential (MP) score at the level of each microbial taxon to predict the effect of each taxon on each metabolite in each sample. Then all the degradation reaction potential scores were subtracted from the synthetic reaction potential scores in the microbial unit to obtain a Community-level metabolic potential (CMP) score. A regression model based on rank prediction was then used to regression the CMP score with the metabolomic test results to assess whether the CMP score significantly predicted metabolite levels. *P* < 0.1 is considered to be a microbial controlled metabolite. Finally, the overall model was decomposed into the contribution of each taxa, and the specific taxa that can affect the change of each metabolite was identified.

## 3 Results

### 3.1 Obesity-prone and obesity-resistant model was successfully established

**Figure 1:**
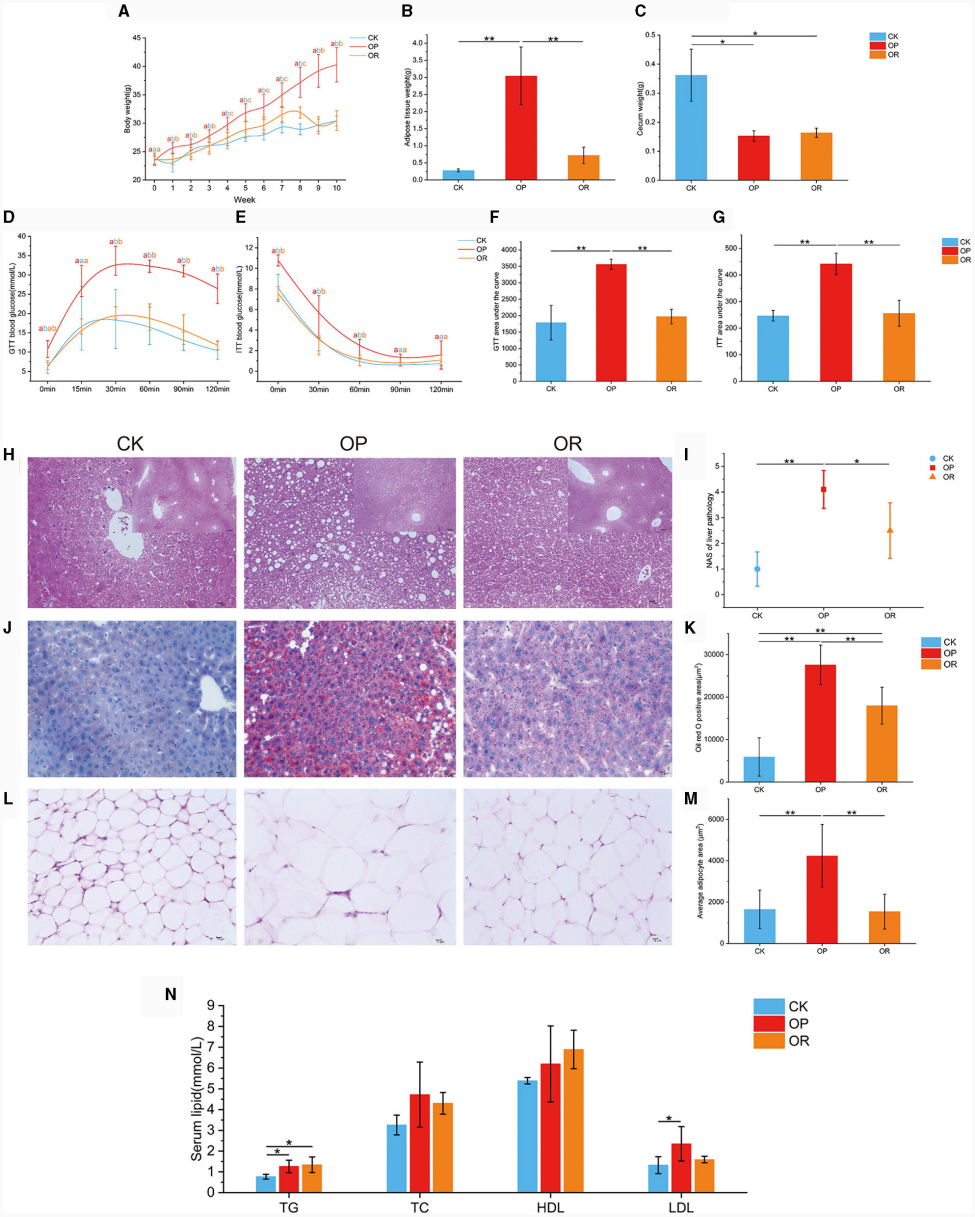
The obesity-prone and obesity-resistance models were successfully established, and there were significant differences in phenotype data among the CK, OP, and OR groups of mice. Body weights of CK, OP and OR groups of mice at 0–10 weeks **(A)**; fat tissue weights **(B)**; cecum weights **(C)**; blood glucose levels in GTT **(D)** and ITT **(E)**; and their corresponding area under the curve: GTT AUC **(F)** and ITT AUC **(G)**. HE staining of liver tissue sections revealed steatosis **(H)**; liver NAS score **(I)**; liver tissue sections stained with oil red O **(J)**; total area of lipid droplets **(K)**; HE staining of adipose tissue slices around the epididymis **(L)**; average area of adipocytes **(M)**; and serum TG, TC, HDL, and LDL levels **(N)**. Single factor ANOVA analysis showed statistical differences (*P* < 0.05). Different lowercase letters indicate significant differences between groups. *P < 0.05 and **P < 0.01.

After high-fat diet consumption, the body weight of OP mice was consistently significantly higher than that of OR and CK mice (*P* < 0.05), while the body weight of OR mice was significantly higher than that of CK mice only from week 4 to week 8 (Figure 1A, *P* < 0.05). Adipose tissue weights were significantly higher in OP mice than in CK and OR mice (Figure 1B, *P* < 0.01). Cecal weights were significantly higher in CK mice than in OP and OR mice (Figure 1C, *P* < 0.05). Compared with CK mice, the blood glucose levels and their corresponding area under curve (AUC) in both insulin tolerance test (ITT) and glucose tolerance test (GTT) of OP mice were significantly higher than those of CK and OR mice (*P* < 0.01), but there was no significant difference between CK and OR mice (Figures 1D–G). This suggests that insulin resistance and glucose tolerance were impaired in OP mice but not in OR mice.

Hematoxylin and eosin (H&E) staining of liver tissue sections revealed extensive steatosis in the livers of OP mice but not in CK and OR mice (Figure 1H). Meanwhile, the liver NAS scores of OP mice were significantly higher than those of CK and OR mice (*P* < 0.05), while there was no significant difference between CK and OR mice (Figure 1I). Oil red O staining of liver tissue sections showed that the total area of lipid droplets in the liver of OP mice was significantly larger than that of CK and OR mice, and that of OR mice was also significantly larger than that of CK mice (Figures 1J, K, *P* < 0.01). H&E staining of periepididymal adipose tissue showed that the mean adipocyte area of OP mice was significantly larger than that of CK and OR mice (Figures 1L, M, *P* < 0.01). Serum TG levels of OP and OR mice were significantly higher than those of CK mice (*P* < 0.05), and serum LDL levels of OP mice were significantly higher than those of CK mice (Figure 1N, *P* < 0.05).

The body weight, adipose tissue weight, glucose tolerance, insulin tolerance, NAS score, and mean adipocyte area of OR mice were not significantly different from those of CK mice, but were significantly different from those of OP mice. This indicates that obesity did not develop in OR mice but did in OP mice. Therefore, we have successfully established obesity-prone and obesity-resistant models.

### 3.2 The gut microbiota of obesity-prone and obesity-resistant mice were significantly different

#### 3.2.1 Alpha diversity analysis and Beta diversity analysis

**Figure 2:**
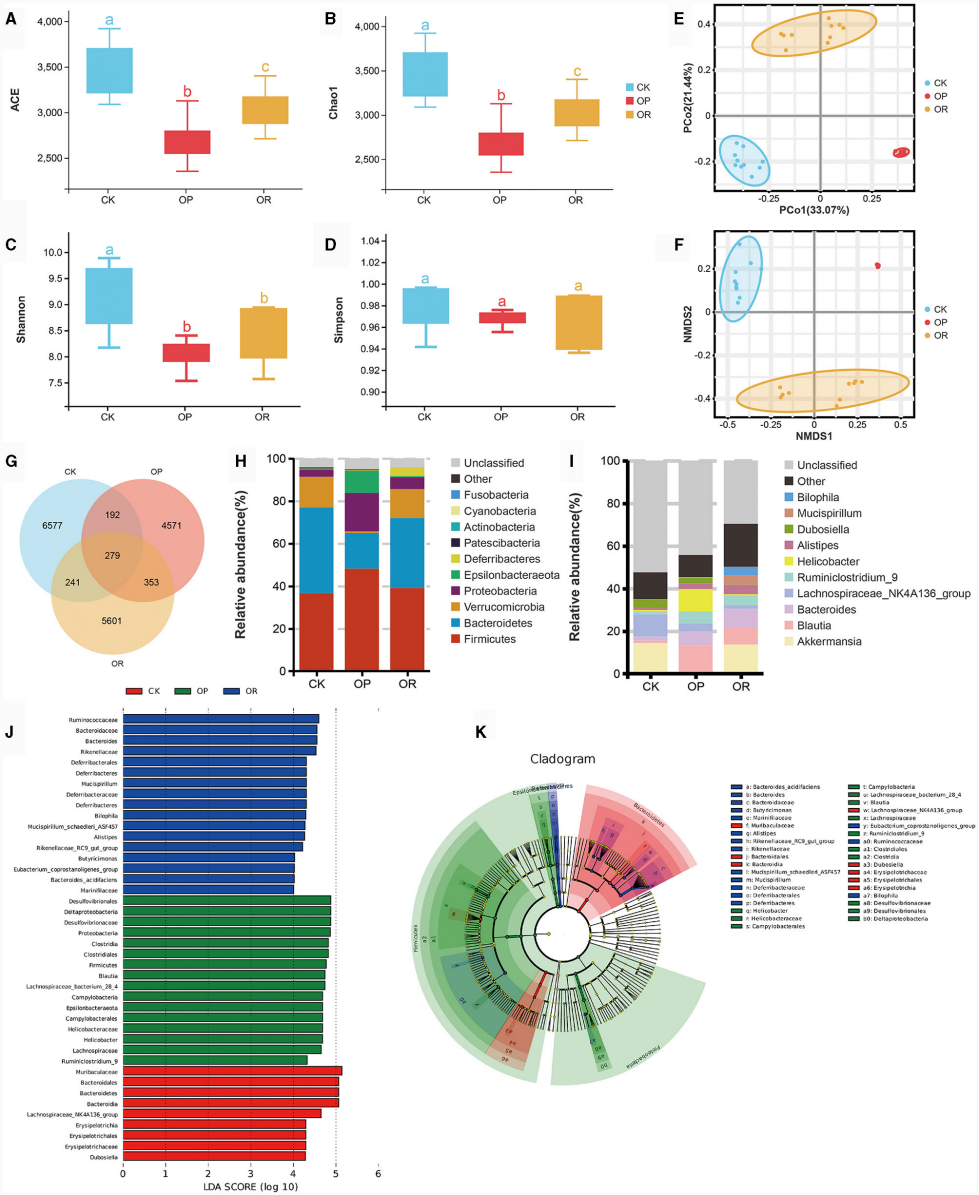
There was a significant difference in gut microbiota between obesity-prone and obesity-resistant mice. Alpha diversity analysis showed Ace **(A)**, Chao1 **(B)**, Shannon **(C)** and Simpson **(D)** values of intestinal microbiota in CK, OP and OR groups of mice; PCoA **(E)** and NMDS **(F)** in Beta diversity analysis; the venn plot showed the number of OTUs **(G)**; stacking diagram of species abundance at phylum level **(H)** and genus level **(I)**; and histogram **(J)** and cadogram **(K)** of LDA values of biomarkers in the LEfSe analysis. In the cadogram, the circles radiating from inside to outside represented the taxonomic level from kingdom to species, each circle at different taxonomic levels represented a species at that taxonomic level, and the size of the circle was proportional to the relative abundance. The *Kruskal-Wallis rank-sum test* showed statistical differences. Different lowercase letters indicate significant differences between groups (*P* < 0.05).

Alpha diversity analysis showed that ACE, Chao1 and Shannon values of gut microbiota of OP and OR mice were significantly lower than those of CK mice (*P* < 0.05), and ACE and Chao1 values of OR mice were significantly higher than those of OP mice (Figures 2A–D, *P* < 0.05). These results show that the species richness and homogeneity of the gut microbiota of OP and OR mice were significantly lower than those of CK mice, and the species richness of OP mice was significantly lower than that of OR mice, but there was no significant difference in species homogeneity between OP mice and OR mice.

In the analysis of Beta diversity, both Principal Co-ordinate Analysis (PCoA) based on linear microbial community structure and Non-metric analysis of microbial structure Multidimensional Scales (NMDS) based on nonlinear microbial structure have shown that the samples of the three groups of mice were well aggregated within the group and the trend of separation between the groups was obvious (Figures 2E, F). This indicates that the gut microbiota structure of mice in each group was highly consistent, and the gut microbiota structure of mice in each group was different.

#### 3.2.2 Species composition analysis and functional prediction

The venn diagram showed that 279 OTUs were shared among the three groups of mice and that 6,577 OTUs were unique to CK mice, with more than 4,571 OTUs being unique to OP mice and 5,601 OTUs being unique to OR mice (Figure 2G). At the phylum level, the gut microbiota of the three groups of mice was mainly composed of *Firmicutes, Bacteroidetes, Verrucomicrobia* and *Proteobacteria* (Figure 2H). At the genus level, the top ten bacteria genera in relative abundance were *Akkermansia, Blautia, Bacteroides, Lachnospiraceae_NK4A136_group, Ruminiclostridium_9, Helicobacter, Alistipes, Dubosiella, Mucispirillum*, and *Bilophila* (Figure 2I).

LEfSe analysis was used to determine the specific bacterial index groups in the three groups, and species with LDA score > 4 are considered biomarkers. At the genus level, the gut microbiota biomarkers of CK mice were *Dubosiella* and *Lachnospiraceae_NK4A136_group*, while the biomarkers of OP mice were *Blautia, Ruminiclostridium_9* and *Helicobacter*. The biomarkers of OR mice were *Bacteroides, Butyricimonas, Alistipes, Rikenellaceae_RC9_gut_group, Mucispirillum, Bilophila* and *Eubacterium_coprostanoligenes_group* (Figures 2J, K). Among the top ten relative abundance bacteria genera, except for *Akkermansia*, all belong to biomarkers. *Akkermansia* was significantly down-regulated in OP mice compared with CK and OR mice (*P* < 0.05), while other biomarkers were significantly up-regulated in their represented groups (Supplementary Figures 1a–m, *P* < 0.05).

Based on 16S rDNA sequencing, we used PICURSt2 to make functional predictions of the gut microbiota. The results show that there were significant differences in the enrichment of functional genes in 124 tertiary metabolic pathways among the three groups of mice.

### 3.3 There were significant differences in gut metabolites between obesity-prone and obesity-resistant mice

#### 3.3.1 Multivariate statistical analysis

In both positive and negative ion modes, PCA, PLS-DA and OPLS-DA results showed that CK mice showed a significant separation trend from OP and OR mice, while OP and OR mice showed no significant separation trend (Supplementary Figures 2a–c). Q2 intercepts of OPLS-DA models established under positive and negative ion modes were all <0, indicating that the models were stable and reliable without overfitting (Supplementary Figure 2d). We identified VIP > 1 and *P* < 0.05 metabolites as differential metabolites. A total of 267 differential metabolites were identified. There were 190 different metabolites between CK and OP mice, 160 different metabolites between CK and OR mice, and 93 different metabolites between OP and OR mice (Supplementary Figure 2e).

#### 3.3.2 Obesity-related differential metabolites

**Figure 3:**
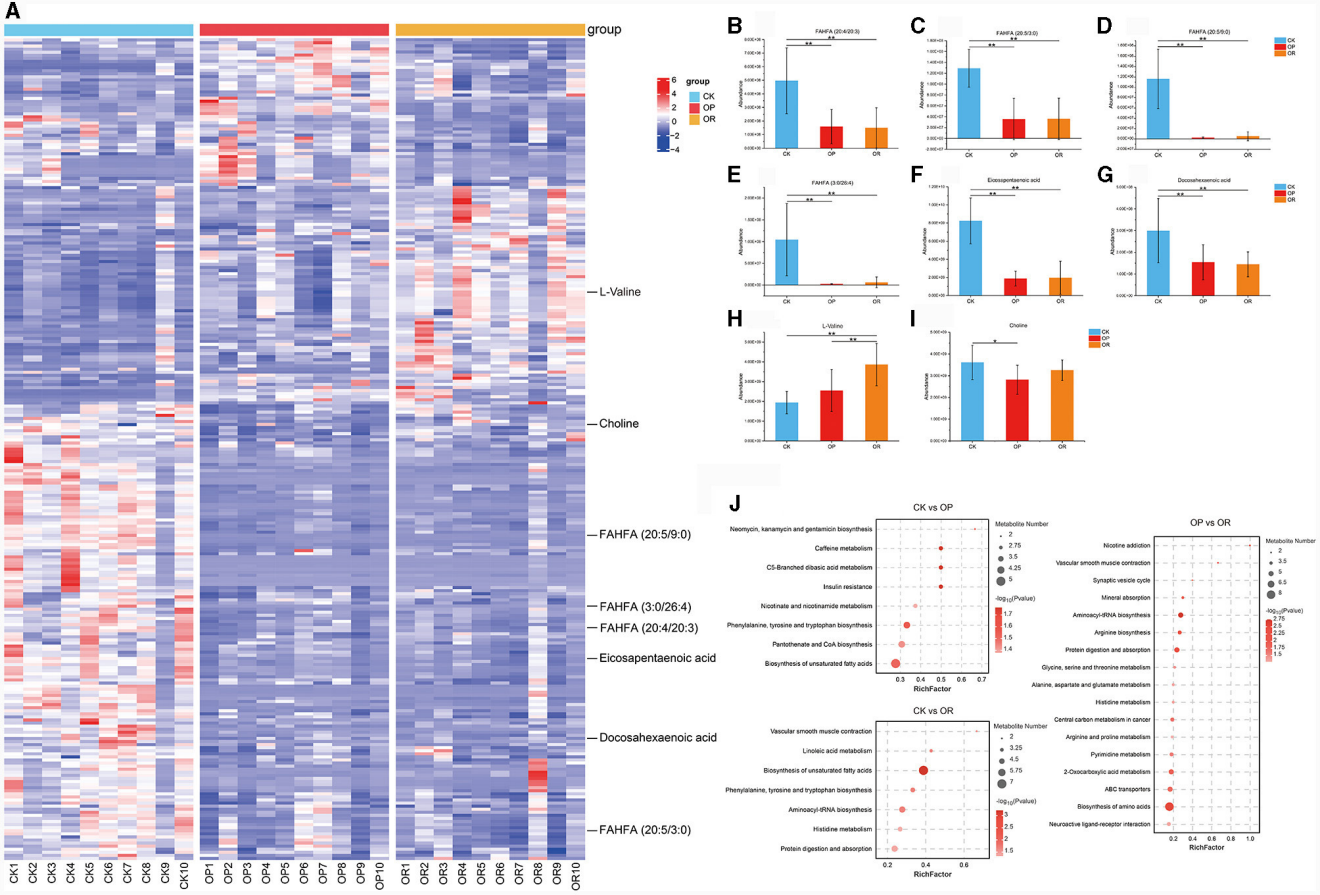
There was a significant difference in gut metabolites between obesity-prone and obesity-resistant mice. Cluster analysis heat map **(A)**; abundance of metabolites FAHFA (20:4/20:3) **(B)**, FAHFA (20:5/3:0) **(C)**, FAHFA (20:5/9:0) **(D)**, FAHFA (3:0/26:4) **(E)**, Eicosapentaenoic acid **(F)**, Docosahexaenoic acid **(G)**, L-valine **(H)** and choline **(I)**; KEGG pathway enrichment analysis **(J)**. The *Kruskal-Wallis rank-sum test* showed statistical differences (*P* < 0.05). “*” indicates *P* < 0.05 and “**” indicates *P* < 0.01.

The cluster heat map reflects the differences in the distribution of metabolites (Figure 3A). In this study, most of the fatty acid esters of hydroxy fatty acids (FAHFAs), which were negatively associated with obesity, were significantly down-regulated in both OP and OR mice compared to CK mice, including FAHFA (20:4/20:3), FAHFA (20:5/3:0), FAHFA (20:5/9:0) and FAHFA (3:0/26:4) (Figures 3B–E, *P* < 0.01). Omega-3 polyunsaturated fatty acids, eicosapentaenoic acid (EPA) and docosahexaenoic acid (DHA) were significantly down-regulated in both OP and OR mice compared to CK mice (Figures 3F, G, *P* < 0.01). L-Valine was significantly up-regulated in OR mice compared with OP and CK mice (Figure 3H, *P* < 0.01). Choline was significantly down-regulated in OP mice compared with CK mice (Figure 3I, *P* < 0.05). In addition, the abundance of various phospholipids, including PE, PC, PI and PG, also differed significantly among the three groups of mice (Supplementary Table 2).

#### 3.3.3 KEGG pathway analysis

To further explore the metabolic pathways affected by the differential metabolites, KEGG pathway enrichment analysis was performed on the differential metabolites. The analysis found that eight and seven KEGG pathways were significantly enriched in OP and OR mice, respectively, as compared to CK mice (Figure 3J, *P* < 0.05). Among them, phenylalanine, tyrosine and tryptophan biosynthesis and biosynthesis of unsaturated fatty acids were both significantly changed in OP and OR mice. Seventeen KEGG pathways were significantly enriched between OP and OR mice, including those involved in protein digestion and absorption and histidine metabolism (Figure 3J, *P* < 0.05).

The above results show that the types, structures and metabolic pathways of the intestinal metabolites of three groups of mice, have significant differences.

### 3.4 Association analysis of gut 16S rDNA sequencing and untargeted metabolome

#### 3.4.1 Correlation analysis between gut microbiota and differential metabolites

**Figure 4:**
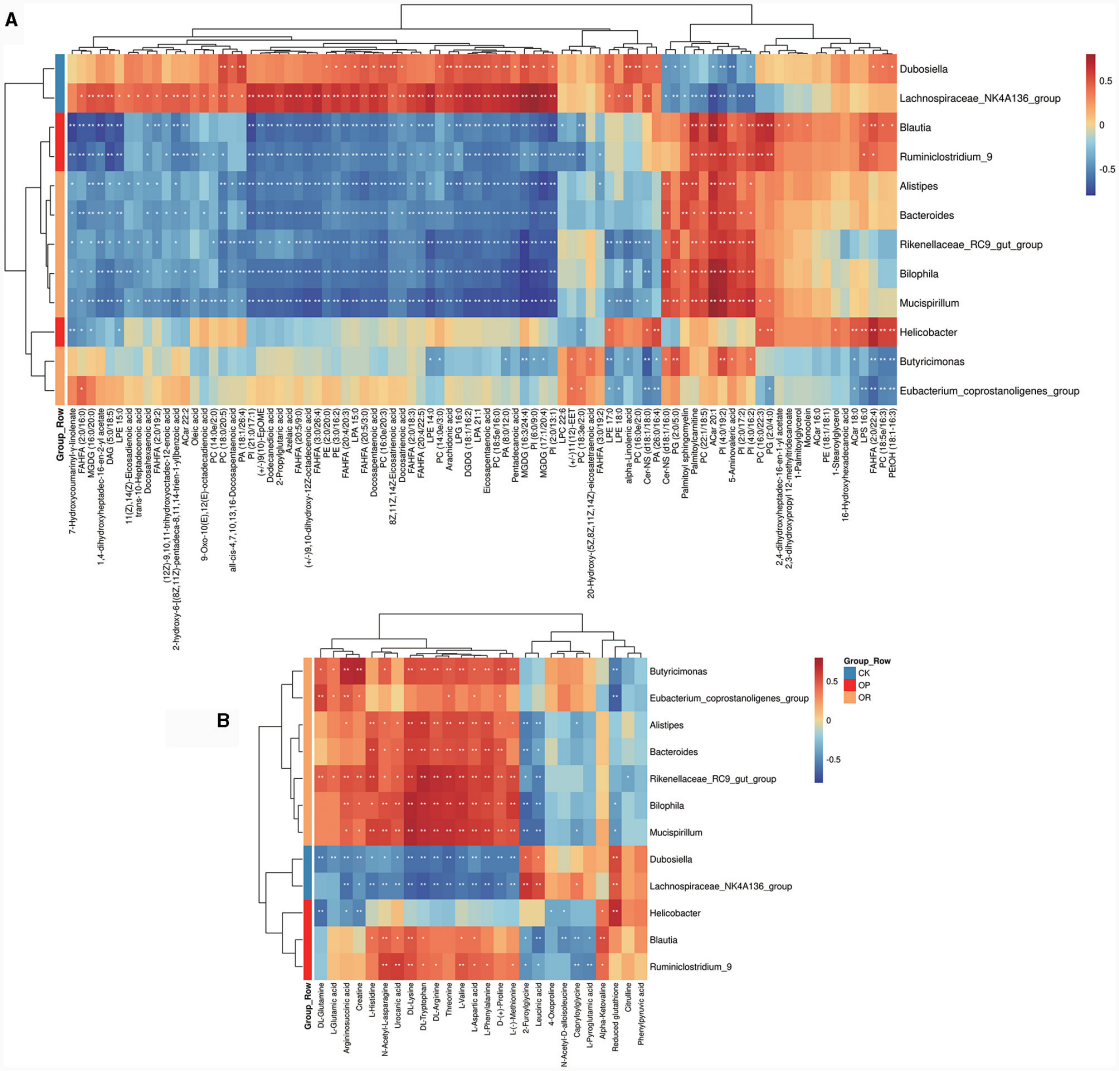
Heat map for correlation analysis between gut biomarkers and differential metabolites. Heat map for fatty acids and their derivatives **(A)** and amino acids and their derivatives **(B)**. The correlation coefficient *r* is shown in color. *r* > 0 represents a positive correlation and is shown in red; *r* < 0 represents a negative correlation and is shown in blue. The darker the color, the stronger the correlation. “*” indicates *P* < 0.05, and “**” indicates *P* < 0.01.

In order to explore the relationship between the changes of metabolites and gut microbiota, 12 biomarkers with LDA > 4 in LEfSe analysis were correlated with all 267 differential metabolites. To make the results easier to observe, we divided the 267 differential metabolites into fatty acids and their derivatives (Figure 4A), amino acids and their derivatives (Figure 4B) and other types of metabolites (Supplementary Figure 3). Results show that FAHFA (20:4/20:3), FAHFA (20:5/3:0), FAHFA (20:5/9:0), FAHFA (3:0/26:4), EPA and DHA, which were all significantly down-regulated in both OP and OR mice, were significantly positively correlated with CK mouse biomarkers *Lachnospiraceae_NK4A136_group*, negatively correlated with OP mouse biomarkers *Blautia* and *Ruminiclostridium_9*, and negatively correlated with OR mouse biomarkers *Rikenellaceae_RC9_gut_group, Alistipes* and *Bilophila* (Figure 4A, *P* < 0.05). In addition, multiple phospholipids including various PE, PC, PI and PG were also significantly associated with biomarkers in LEfSe analysis (Figure 4A, *P* < 0.05).

#### 3.4.2 MIMOSA analysis

**Figure 5:**
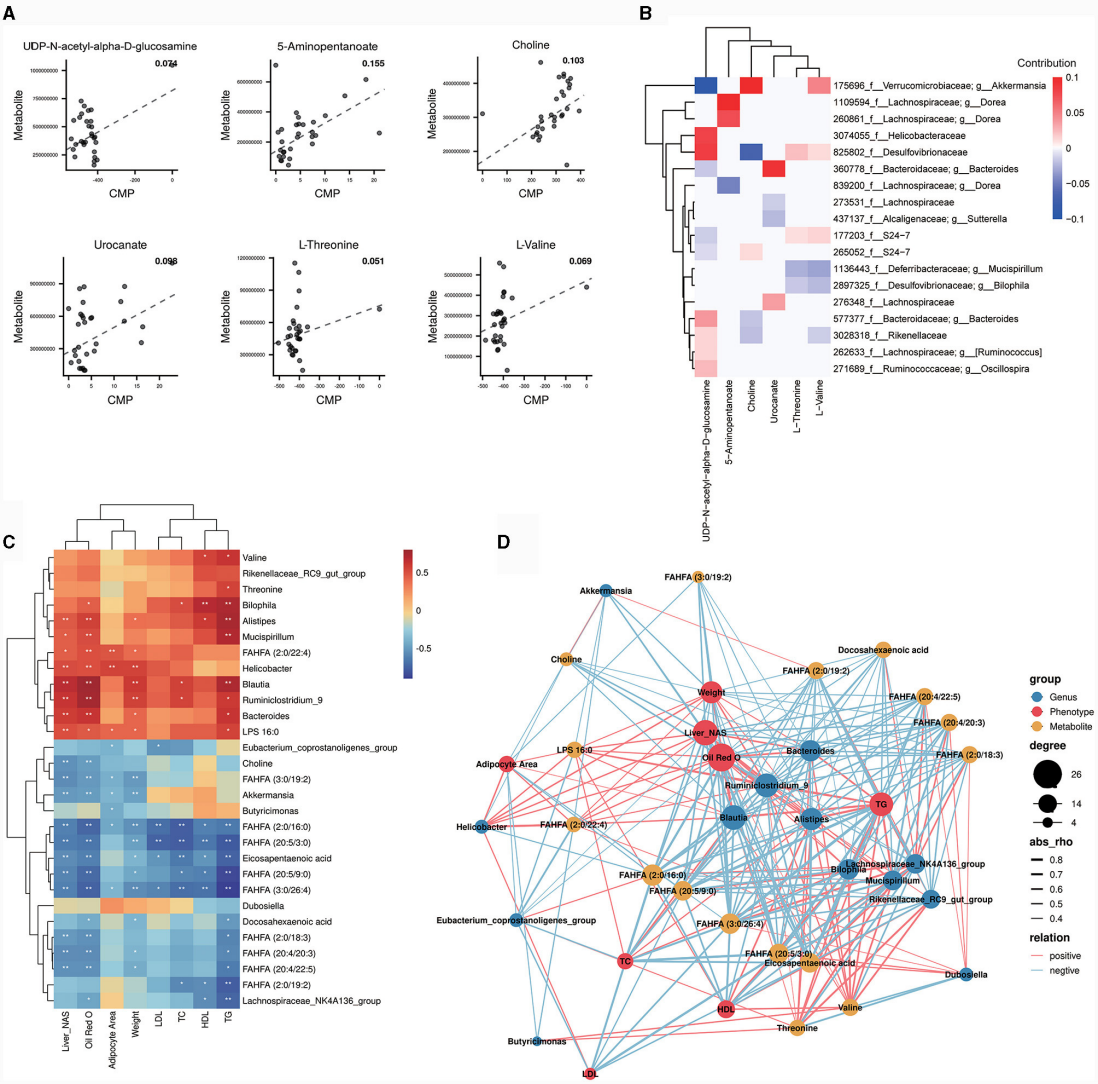
MIMOSA analysis and correlation analysis between bacterial genera, differential metabolites and phenotypes. Scatter plot of regression analysis between CMP values of six metabolites and actual abundance **(A)**; heat map of contribution values of each taxonomic unit to changes in these six metabolites **(B)**; heat map of correlation analysis between bacterial genera and some differential metabolites with phenotype **(C)**. The correlation coefficient *r* is shown in color. *r* > 0 represents a positive correlation and is shown in red; *r* <0 represents a negative correlation and is shown in blue. The darker the color, the stronger the correlation. “*” indicates *P* < 0.05 and “**” indicates *P* < 0.01. Network diagram for correlation analysis of bacterial genera, metabolites and obesity related phenotypes **(D)**. The size of a node represents the number of connected nodes, and the thickness of a line represents the size of the correlation coefficient r. r>0 represents a positive correlation and is shown in red; *r* < 0 represents a negative correlation and is shown in blue.

To further explore the role of gut microbiota on differential metabolites, a regression analysis based on database to predict microbial metabolic potential, namely MIMOSA analysis, was used. The results show that there was a significant regression relationship between the CMP values of six metabolites and the abundance of these metabolites detected in the metabolome (*P* < 0.1). These six metabolites were UDP-N-acetyl-alpha-D-glucosamine, 5-Aminopentanoate, choline, urocanate, L-Threonine and L-Valine (Figure 5A). This suggests that changes in the abundance of these six metabolites are regulated by the gut microbiota. Subsequently, we decomposed the overall model into the contribution of each taxon. The results showed that *g_Akkermansia* (Greengene ID: 175696) could produce glycerophosphoryl diester phosphodiesterase (glpQ) to synthesize choline (KEGG ID: C00114) and valyl-tRNA synthetase (VARS) to degrade L-Valine (KEGG ID: C00183) and it positively contributed to the changes in choline (KEGG ID: C00114) and L-Valine (KEGG ID: C00183) (Figure 5B). In addition, the abundance of *g_Akkermansia* (Greengene ID: 175696) was also significantly down-regulated in OP mice as compared to CK and OR mice in MIMOSA analysis (Supplementary Figure 1n, *P* < 0.05). This suggests that down-regulation of *g_Akkermansia* (Greengene ID: 175696) abundance leads to down-regulation of choline (KEGG ID: C00114) and up-regulation of L-Valine (KEGG ID: C00183).

#### 3.4.3 Correlation analysis of gut microbiota, differential metabolites and obesity-related phenotypes

The biomarkers from LEfSe analysis, the top 10 genera in relative abundance and some differential metabolites were correlated with obesity-related phenotypes. The results showed that FAHFA (20:4/20:3), FAHFA (20:5/3:0), FAHFA (20:5/9:0), FAHFA (3:0/26:4), DHA and EPA, which were significantly down-regulated in both OP and OR mice, were all significantly negatively correlated with liver NAS scores, oil red O staining positive area, body weight and serum TG (Figure 5C, *P* < 0.05). Notably, *Akkermansia* and choline, which were both significantly down-regulated in OP mice, were both significantly negatively correlated with liver NAS score and oil red O staining positive area (Figure 5C, *P* < 0.05). In addition, *Akkermansia* was significantly negatively correlated with adipocyte area and body weight (Figure 5C, *P* < 0.05). The correlations among genus, metabolite and obesity-related phenotypes were visualized by the correlation network map. Among them, *Akkermansia* was significantly positively correlated with choline, while both *Akkermansia* and choline were significantly negatively correlated with obesity-related phenotypes (Figure 5D). The above results suggest that the effect of *Akkermansia* on choline may be a key factor in the influence of gut microbiota on obesity susceptibility.

#### 3.4.4 Correlation analysis between PICURSt2 function prediction and metabolic pathways

**Figure 6:**
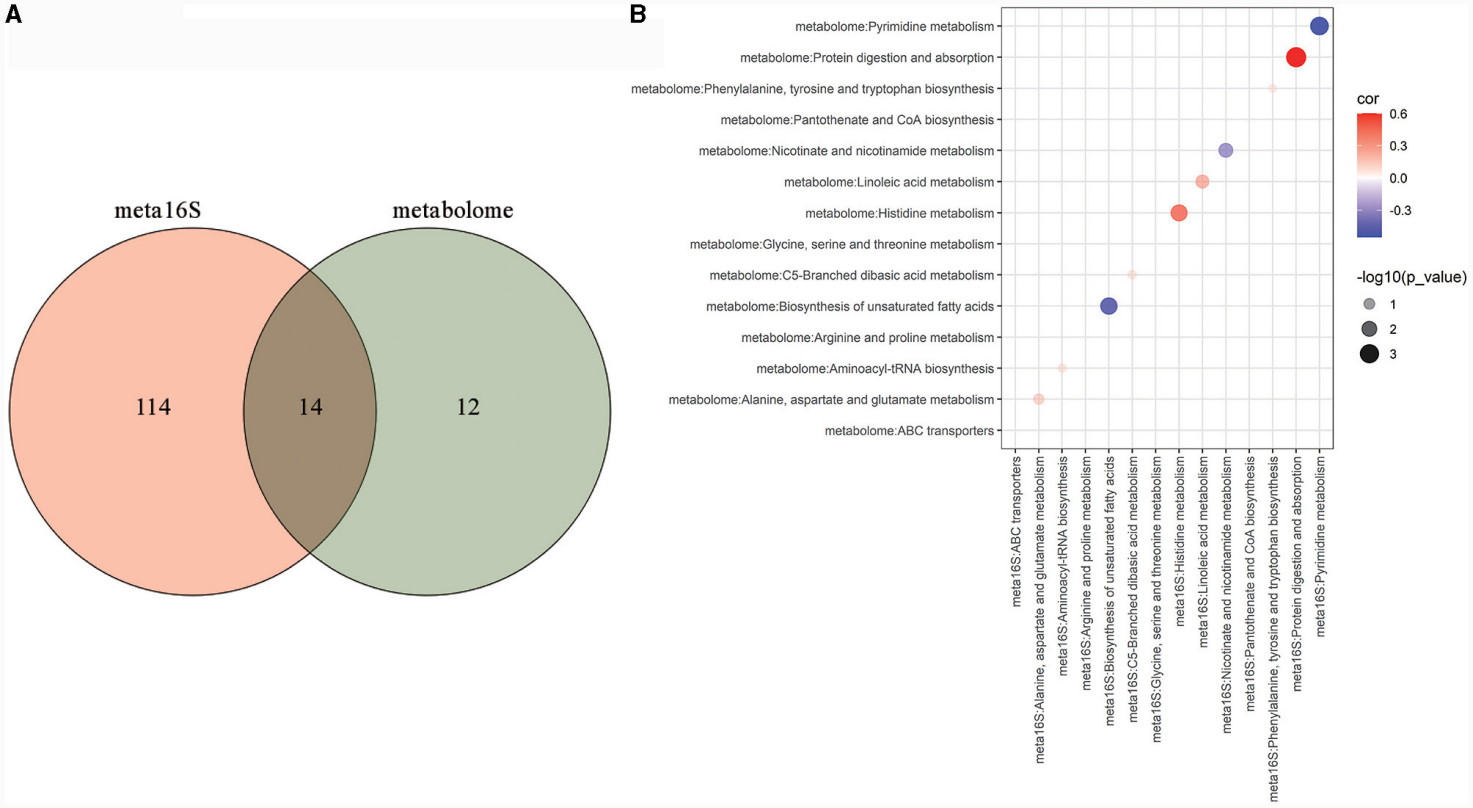
Metabolic pathways that may be affected by gut microbiota. Venn analysis between KEGG metabolic pathways with significant differences in PICURSt2 functional prediction and KEGG metabolic pathways significantly enriched in the metabolome **(A)**; bubble plot of the correlation between the predicted functional gene abundance of the 14 crossed pathways in Venn analysis and the total abundance of all metabolites in the pathways **(B)**.

To further explore the metabolic pathways that may be affected by gut microbiota, correlation analysis was performed on metabolic pathways that were significantly different in PICURSt2 function prediction and metabolic pathways that were significantly enriched in the metabolome. The venn diagram showed that 14 metabolic pathways were significantly different in PICURSt2 function prediction and were also significantly enriched in the metabolome (Figure 6A). We correlated the predicted functional gene abundance of these 14 metabolic pathways with the total abundance of all metabolites in the pathway. The results show that the abundances of functional genes for histidine metabolism, linoleic acid metabolism and protein digestion and absorption were significantly positively correlated with the degree of metabolite enrichment (Figure 6B, *P* < 0.05). This suggests that histidine metabolism, linoleic acid metabolism and protein digestion and absorption may be affected by gut microbiota.

## 4 Discussion

### 4.1 Establishment of obesity-prone and obesity-resistant models

In our study, mice weighing more than 1.2 times the average weight of CK mice were considered susceptible to obesity, while those weighing <1.1 times the average weight of CK mice were considered resistant to obesity. In the actual experiments, the number of obese-prone mice and obese-resistant mice were both approximately one-third of the total, which is consistent with the findings of previous studies conducted by Zhang et al. (2018) and Gu et al. (2019). In addition, there were significant differences in many obesity-related phenotypes between OP and OR mice, suggesting that the obesity-prone and obesity-resistant models were successfully established.

Compared with OR mice, OP mice showed significant insulin resistance. This may be due to higher levels of circulating fatty acids and systemic inflammation, including fat and intestinal inflammation, in OP mice than in OR mice. As a result, OP mice exhibit higher serine phosphorylation of insulin receptor substrate 1 (IRS-1) and more severe insulin signaling blocking (Johnson and Olefsky, 2013; Ardiansyah et al., 2018). In addition, different metabolites produced by the gut microbiota of OP and OR mice, such as SCFAs and bile acids, enter the circulation and pass through G-protein coupled receptors (GPCRs), nuclear hormone receptors or host proteins post-translational modifications (such as lysine acetylation), thereby affecting whole-body insulin sensitivity (Johnson and Olefsky, 2013).

### 4.2 Effects of gut microbiota on obesity

In recent years, *Akkermansia*, especially *Akkermansia muciniphila*, has been generally recognized as being negatively associated with obesity and insulin resistance (Dao et al., 2016; Hasani et al., 2021). For the top ten genera in relative abundance, all the genera except *Akkermansia* were biomarkers for each group. Among them, *Lachnospiraceae_NK4A136_group*, a biomarker of the CK group, has been shown to produce butyrate, increase the anti-inflammatory factor IL-10 and have a positive anti-inflammatory effect in obese mice (Hu et al., 2019). In addition, *Lachnospiraceae_NK4A136_group* also contributes to weight loss (Wang et al., 2020) and improves insulin sensitivity through spermidine (Ma et al., 2020; Zhao et al., 2021). Among the biomarkers in the OP group, both *Blautia* and *Ruminiclostridium_9* have been found to promote obesity. *Blautia* is known to contribute to promoting obesity-related metabolic diseases such as hypertriglyceridemia, fatty liver disease and insulin resistance (Zeng et al., 2019). Additionally, studies have shown that *Blautia* can catalyze the synthesis of deoxycholic acid (DCA) by synthesizing 7α-dehydroxylase (Lin et al., 2019). Due to its high hydrophobicity, high levels of DCA in the gut may disrupt the integrity of the intestinal epithelial barrier (Stenman et al., 2013). *Ruminiclostridium_9* is widely considered to be a high-fat diet-dependent genus (Hou et al., 2021; Li et al., 2022b; Zhao et al., 2022) and is significantly positively correlated with serum LPS levels (Zhao et al., 2021; Lan et al., 2023). Among the biomarkers of the OR group, *Alistipes* and *Rikenellaceae_RC9_gut_group* have been shown to be significantly negatively correlated with body weight and adipose tissue weight, and they may play a key role in the prevention of obesity (Fu et al., 2022). In previous studies, the role of *Bilophila* in the development of obesity has been debated. In some studies, *Bilophila* has been identified as a potentially harmful genus producing LPS and reducing intestinal sulfate (Dostal Webster et al., 2019). However, in other studies, *Bilophila* was significantly negatively correlated with HFD-induced multi-tissue metabolic disorders and may play an important role in the process of α-Linolenic acid improving multi-tissue homeostasis in HFD mice (Dostal Webster et al., 2019).

### 4.3 Effect of gut microbiota on the susceptibility to obesity through gut metabolites

Branched fatty acid esters of hydroxy fatty acids (FAHFAs) are composed of one molecule of fatty acid (FA) and one molecule of hydroxy fatty acid (HFA) linked by an ester bond. Among them, FAHFAs (16:0/18:0), specifically palmitic acid esters of hydroxy stearic acids (PAHFAs), particularly 9-PAHFAs, have been shown to improve glucose tolerance and reduce adipose tissue inflammation in high-fat diet mice (Brejchova et al., 2020). In addition, polyunsaturated FAHFAs have a stronger anti-inflammatory function relative to PAHFAs (Brejchova et al., 2020). FAHFA (20:5/n_1_:n_2_) and FAHFAs containing eicosapentaenoic acid (EPA) can increase the expression of NrF2-dependent antioxidant enzymes in HCC cells (B Gowda et al., 2020). It has also been shown that *Bacteroides acidifaciens* may improve glucose homeostasis and inflammation in mice through FAHFAs (Yan et al., 2023). In our study, FAHFA (20:4/20:3), FAHFA (20:5/3:0), FAHFA (20:5/9:0) and FAHFA (3:0/26:4), all of which belong to the polyunsaturated FAHFAs, were significantly down-regulated in OP and OR mice compared to the CK group. All of them were significantly positively correlated with *Lachnospiraceae_NK4A136_group* and significantly negatively correlated with most of the OP and OR group biomarkers. Additionally, these four FAHFAs were also significantly negatively correlated with phenotypic measures of obesity. This suggests that HFD-induced gut microbiota may promote obesity by down-regulating FAHFAs.

As omega-3 polyunsaturated fatty acids (ω-3 PUFAs), EPA and DHA can activate AMPK to promote the β-oxidation of fatty acids and can also act on PPARγ to inhibit lipogenesis (D'Angelo et al., 2020; Fu et al., 2021). Additionally, EPA and DHA can improve insulin sensitivity by up-regulating plasma adiponectin (D'Angelo et al., 2020). Other studies have shown that ω-3 PUFAs can improve gut microbiota imbalance and the impaired gut immune function caused by obesity, and gut microbiota can also reverse the effects on the absorption and metabolism of ω-3 PUFAs (Fu et al., 2021). In our study, both EPA and DHA were significantly down-regulated in OP and OR mice compared to CK mice. However, there was no difference between OP and OR mice. EPA and DHA were also significantly positively correlated with biomarkers in the CK group. They were significantly negatively correlated with most biomarkers in the OP group and the OR group, as well as significantly negatively correlated with obesity-related phenotypes. This suggests that the significant down-regulation of gut EPA and DHA after a high-fat diet may be caused by the gut microbiota induced by high-fat diet. Down-regulated EPA and DHA may contribute to obesity, but do not affect the generation of differences in susceptibility to obesity, similar to FAHFAs.

Phosphoethanolamine (PE), phosphocholine (PC), phosphoinositol (PI), and phosphoglycerol (PG) are the main lipid components found in bacterial membranes (Brown et al., 2023). These bacterial phospholipids are highly diverse, and bacteria can adapt to different environments by changing the length and unsaturation of the acyl chain and adding different groups (such as adding ethanolamine, choline, inositol, or glycerol) to produce PE, PC, PI, or PG, respectively (Zhang and Rock, 2008; Brown et al., 2023). Furthermore, in the mouse gut, gut microbiota has been shown to influence the phospholipid levels of host gut cells (Manca et al., 2020). Alterations in membrane phospholipid levels in the host gut can increase intestinal permeability, resulting in a range of systemic effects, including obesity (de La Serre et al., 2010; Ammendolia et al., 2021). In our study, a variety of phospholipids, including PE, PC, PI and PG, all changed significantly after a high-fat diet. Additionally, these phospholipids were also significantly associated with multiple biomarkers in the LEfSe analysis. This suggests that a high-fat diet may induce obesity by altering phospholipid levels in the gut microbiota.

Branched chain amino acids (BCAA) are thought to be positively associated with obesity and insulin resistance (Yuan et al., 2023). However, it is important to note that in most studies, the increase in BCAA levels is only a consequence of obesity, rather than the cause. It has been demonstrated that glucose metabolism is not impaired, but rather improved in obese patients after additional BCAA supplementation (Woo et al., 2019; Simonson et al., 2020). As a BCAA, valine, when combined with TDCA, can inhibit the release of hypothalamic MCH in obese mice, thereby reducing appetite, body weight and improving glucose tolerance (Quante et al., 2021). Other studies have shown that valine supplementation alone can improve insulinemia induced by a high-fat diet, reduce intrahepatic lipid accumulation and improve lipid metabolism by inhibiting SREBF1 and activating PPARGC1A, ACOX1 and AMPK (Gart et al., 2022). Additionally, the metabolism of valine is also affected by the gut microbiota. Studies have shown that *Akkermansia muciniphila* can significantly affect the level of valine in the culture medium when mucin reaches a certain concentration (Liu et al., 2021). In our experiments, L-Valine was significantly up-regulated in OR mice compared to CK and OP mice. MIMOSA2 analysis revealed that *g_Akkermansia* (Greengene ID: 175696) was capable of producing VARS to degrade L-Valine and positively contributed to the change in L-Valine. Therefore, *g_Akkermansia* (Greengene ID: 175696) could contribute to differences in obesity susceptibility by affecting L-Valine levels after a high-fat diet.

Numerous studies have shown that excessive dietary choline intake can be metabolized to trimethylamine (TMA) by gut microbiota, resulting in increased circulating TMAO levels (Yoo et al., 2021). TMAO has been shown to be positively correlated with obesity (Dehghan et al., 2020). However, it should be noted that in our study, choline levels in OP mice were significantly down-regulated compared with CK mice on a normal diet. Therefore, OP mice are in a state of choline deficiency, rather than choline excess. Studies have shown that choline is significantly up-regulated in obese insulin-sensitive individuals compared to obese insulin-resistant individuals (Al-Sulaiti et al., 2019), which is similar to our results. Additionally, PC synthesis in the liver is mainly dependent on choline intake, and the lack of PC can limit VLDL secretion (Noga and Vance, 2003). Abnormally secreted VLDL can lead to abnormal triglyceride secretion, which causes hepatic steatosis (Corbin and Zeisel, 2012). In addition, inhibition of PC synthesis also leads to increased lipogenesis (Su et al., 2022). Other studies have shown that *Haemophilus influenzae* can produce and use glycerophosphoryl diester phosphodiesterase (glpQ) to remove choline from glycerophosphoryl phosphocholine (Fan et al., 2001). In our study, MIMOSA2 analysis showed that *g_Akkermansia* (Greengene ID: 175696) was able to produce glpQ to synthesize choline and positively contribute to changes in choline levels. This suggests that *g_Akkermansia* (Greengene ID: 175696) can promote obesity proneness by reducing choline levels through glpQ production after high-fat diet.

Studies have shown that gut microbiota can participate in the development of obesity through histidine metabolism, linoleic acid metabolism and protein digestion and absorption (Zhang et al., 2019; Xie et al., 2021; Li et al., 2022a; Ning et al., 2022; Guo et al., 2023). In our study, the abundances of functional genes for histidine metabolism, linoleic acid metabolism and protein digestion and absorption were significantly positively correlated with the degree of metabolite enrichment. This suggests that, at the pathway level, gut microbiota may regulate histidine metabolism, linoleic acid metabolism and protein digestion and absorption to regulate the difference in obesity and obesity susceptibility after a high-fat diet.

## 5 Conclusion

Gut microbiota and gut metabolites were significantly different among the CK, OP, and OR mice. The consumption of a high-fat diet induces alterations in the gut microbiota, resulting in perturbations of EPA, DHA, various FAHFAs, and diverse phospholipids, thereby facilitating the development of obesity. *G_Akkermansia* (Greengene ID: 175696) may contribute to the difference in obesity susceptibility through the synthesis of glpQ, which promotes choline production and the synthesis of VARS, which promotes L-Valine degradation. Additionally, gut microbiota may affect obesity and obesity susceptibility through histidine metabolism, linoleic acid metabolism and protein digestion and absorption pathways. In conclusion, the gut microbiota has the potential to exert an impact on the susceptibility to obesity in mice by means of gut metabolites.

## Data availability statement

The datasets presented in this study can be found in online repositories. The names of the repository/repositories and accession number(s) can be found below: https://www.ncbi.nlm.nih.gov/, PRJNA1013600.

## Ethics statement

The animal studies were approved by Animal Ethics Committee of Southwest Medical University. The studies were conducted in accordance with the local legislation and institutional requirements. Written informed consent was obtained from the owners for the participation of their animals in this study.

## Author contributions

YWe: Conceptualization, Data curation, Formal analysis, Methodology, Visualization, Writing – original draft, Writing – review & editing. YL: Data curation, Formal analysis, Investigation, Methodology, Validation, Visualization, Writing – original draft, Writing – review & editing. HQ: Data curation, Investigation, Visualization, Writing – original draft. BC: Data curation, Investigation, Writing – review & editing. JH: Data curation, Investigation, Writing – review & editing. SL: Data curation, Validation, Writing – original draft. YWa: Conceptualization, Data curation, Investigation, Writing – review & editing. JL: Conceptualization, Data curation, Writing – review & editing. LT: Data curation, Writing – review & editing. BY: Data curation, Writing – review & editing. KL: Data curation, Writing – review & editing. LH: Project administration, Supervision, Writing – review & editing. MH: Project administration, Supervision, Writing – review & editing. QY: Project administration, Supervision, Writing – review & editing. ZY: Project administration, Supervision, Writing – review & editing. WX: Project administration, Supervision, Writing – review & editing. MZ: Project administration, Supervision, Writing – review & editing. XZ: Funding acquisition, Project administration, Validation, Writing – review & editing. RL: Funding acquisition, Project administration, Validation, Writing – review & editing. CG: Conceptualization, Formal analysis, Funding acquisition, Project administration, Supervision, Writing – review & editing.
