# Supplementary figures and images for: Gut microbiota affects obesity susceptibility in mice through gut metabolites
Source: Front Microbiol. 2024 Feb 21;15:1343511. doi: 10.3389/fmicb.2024.1343511 (PMC10916699; doi:10.3389/fmicb.2024.1343511)

# Supplement Figure 1

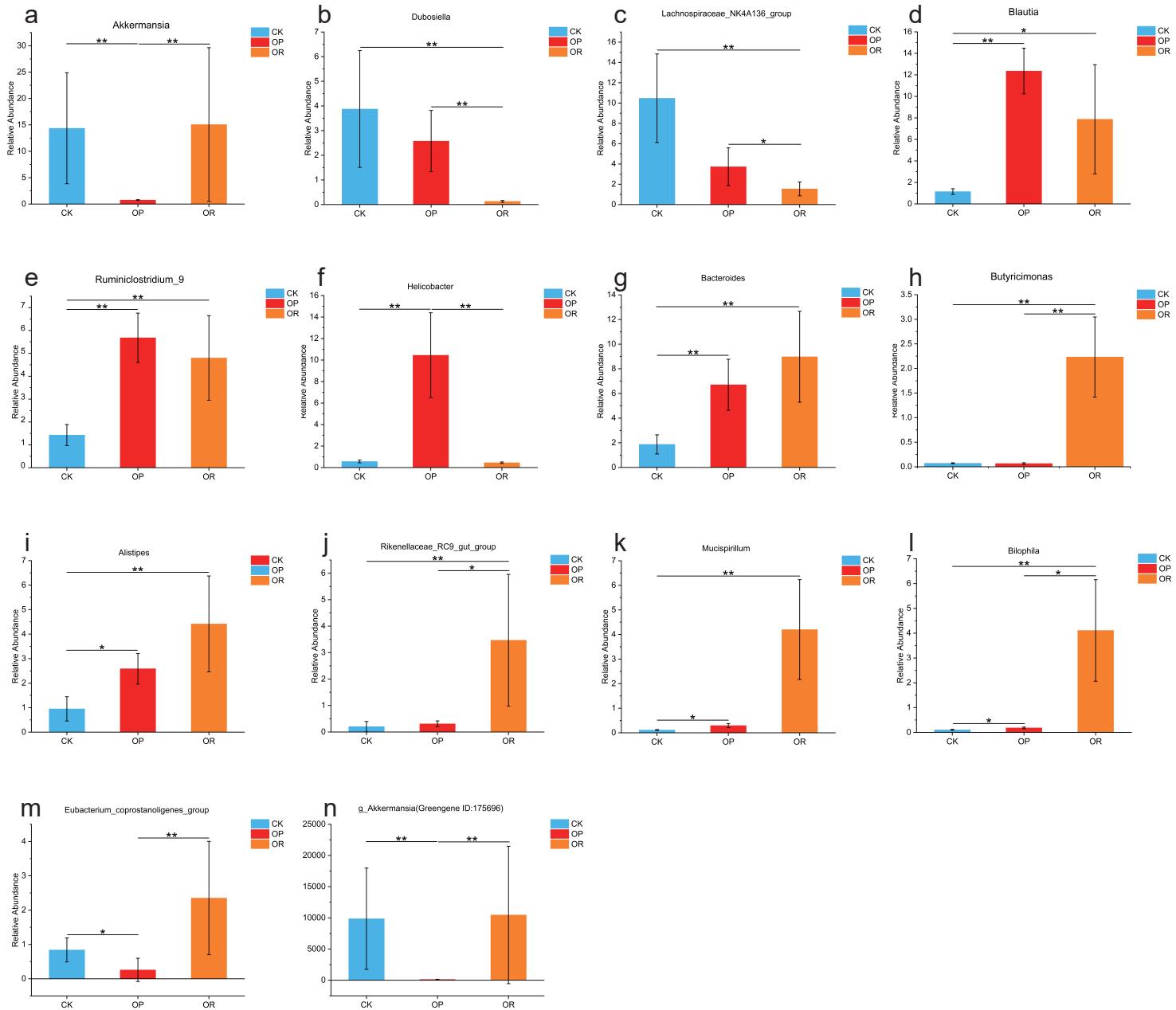

Supplement: Supplementary file 1 [file Data_Sheet_1.PDF]

# Supplement Figure 2

Positive

Negative

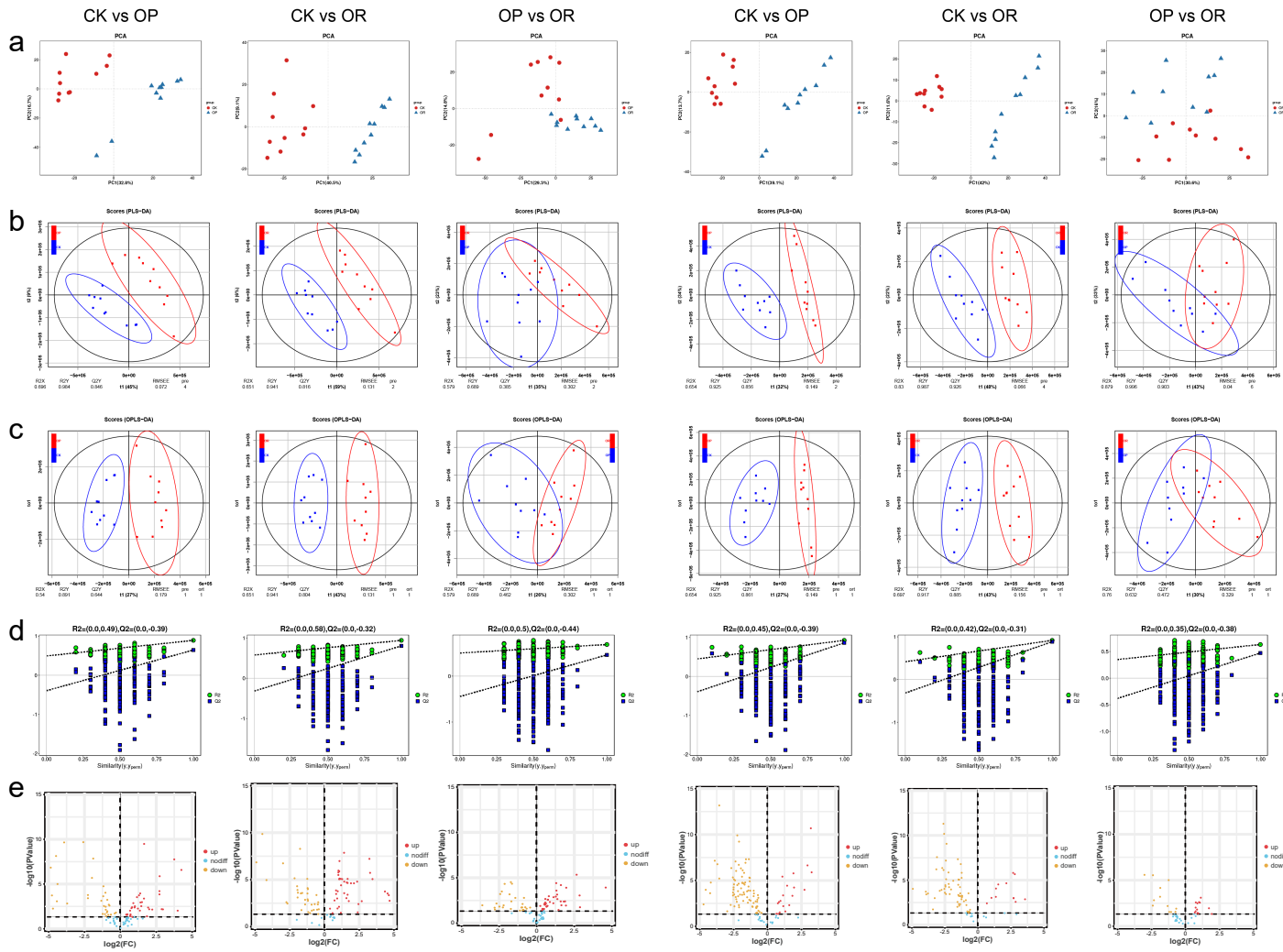

Supplement: Supplementary file 2 [file Data_Sheet_2.PDF]

## Supplement Figure 3

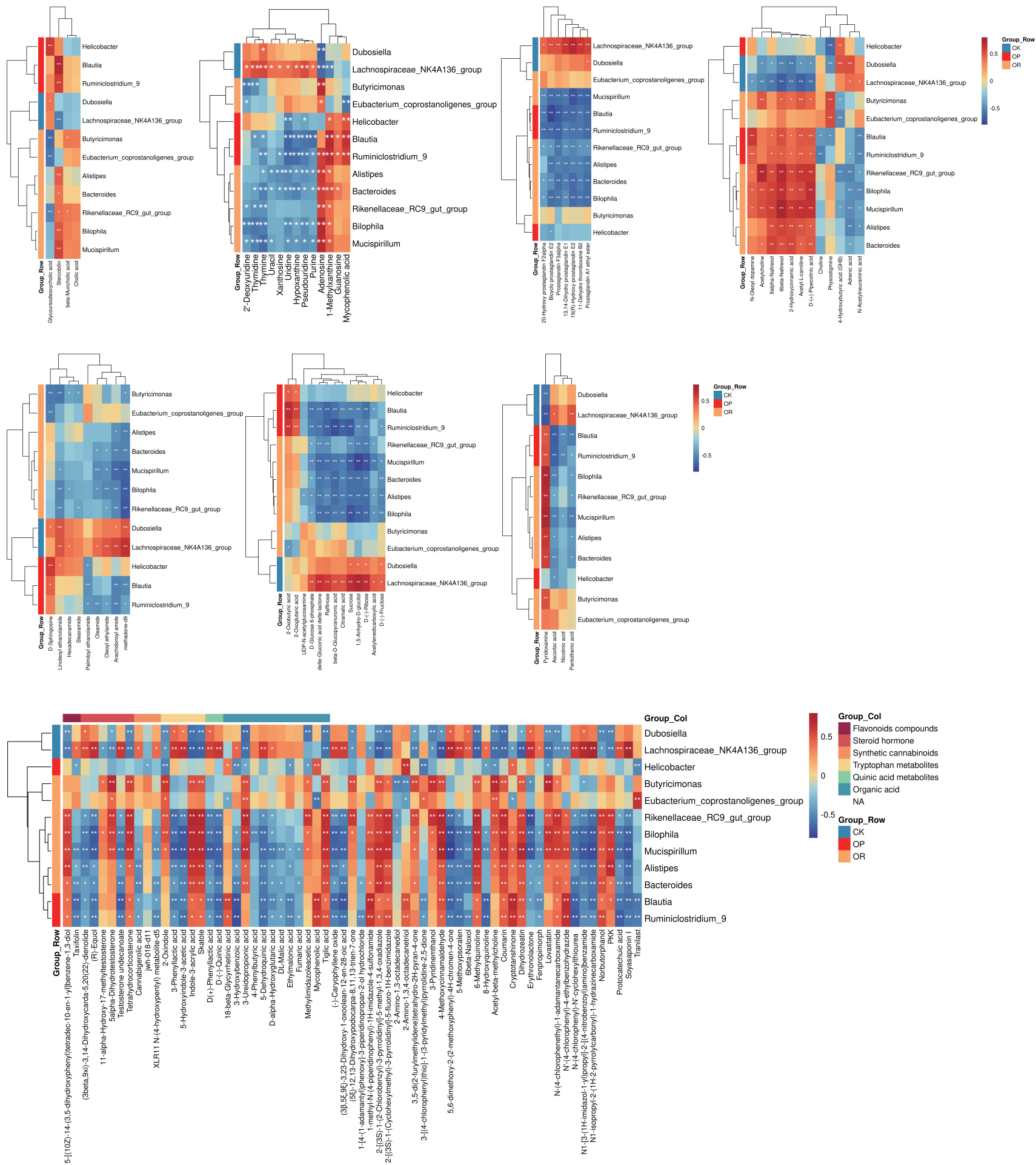

Supplement: Supplementary file 3 [file Data_Sheet_3.PDF]
