# Supplementary material for: Gut microbiota affects obesity susceptibility in mice through gut metabolites
Source: Front Microbiol. 2024 Feb 21;15:1343511. doi: 10.3389/fmicb.2024.1343511 (PMC10916699; doi:10.3389/fmicb.2024.1343511)
Supplement: Supplementary file 4 [file Data_Sheet_4.PDF]

Supplementary Table 1: The detail composition of the normal diet and HFD diet

Normal diet		high-fat diet		
Ingredients	Grams	Class description	Ingredients	Grams
Water	⩽100g	Protein	Casein,Lactic,30 Mesh	200.00g
Crude protein	⩾200g	Protein	Cystine, L	3.00 g
Crude fat	⩾40g	Carbohydrate	Lodex 10	125.00 g
Crude fiber	⩽50g	Carbohydrate	Sucrose,Fine Granulated	72.80 g
Crude ash	⩽80g	Fiber	Solka Floc, FCC200	50.00g
Calcium	10-18g	Fat	Lard	245.00 g
Total phosphorus	6-12g	Fat	Soybean Oil, USP	25.00 g
Lysine	⩾13.2g	Mineral	S10026B	50.00 g
Methionine + Cystine	⩾7.8g	Vitamin	Choline Bitartrate	2.00 g
Arginine	⩾11.0g	Vitamin	V10001C	1.00 g
Histidine	⩾5.5g	Dye	Dye, Blue FD&C #1,Alum.Lake 35-42%	0.05 g
Tryptophan	⩾2.5g			
phenylalanine+Tyrosine	⩾13.0g			
Threonine	⩾8.8g			
Leucine	⩾17.6g			
Isoleucine	⩾10.3g			
Valine	⩾11.7g			
Magnesium	⩾2.0g			
Potassium	⩾5.0g			
Sodium	⩾2.0mg			
Ferrum	⩾120 mg			
Manganese	⩾75mg			
Copper	⩾10mg			
Zinc	⩾30mg			
Iodine	⩾0.5mg			
Selenium	⩾0.1-0.2mg			
Vitamin A	⩾14000 IU			
Vitamin D	⩾1500 IU			
Vitamin E	⩾120 IU			
Vitamin K	⩾5.0mg			
Vitamin B1	⩾13 mg			
Vitamin B2	⩾12mg			
Vitamin B6	⩾12mg			
Niacin Turns	⩾60mg			
Pantothenic Acid	⩾24mg			
Folic Acid	⩾6.00mg			
Biotin	⩾0.20mg			
Vitamin B12	⩾0.022mg			
Choline	⩾1250mg			
