# Supplementary material for: Gut microbiota affects obesity susceptibility in mice through gut metabolites
Source: Front Microbiol. 2024 Feb 21;15:1343511. doi: 10.3389/fmicb.2024.1343511 (PMC10916699; doi:10.3389/fmicb.2024.1343511)
Supplement: Supplementary file 5 [file Data_Sheet_5.PDF]

Compound	CK	OP	OR
PA (18:1/26:4)	6.52E+08±1.88E+08[a]	5.10E+08±1.86E+08[ab]	4.07E+08±1.54E+08[b]
PA (26:0/16:4)	1.54E+08±6.89E+07[ab]	4.47E+08±2.90E+08[a]	8.17E+07±8.83E+07[b]
PA (8:0/12:0)	3.46E+08±1.62E+08[a]	9.57E+07±9.92E+07[b]	6.25E+07±8.24E+07[b]
PC (14:0e/2:0)	1.89E+09±8.68E+08[a]	1.28E+09±5.03E+08[a]	1.17E+09±5.44E+08[a]
PC (14:0e/3:0)	7.30E+08±4.18E+08[a]	4.53E+08±2.70E+08[ab]	3.18E+08±2.30E+08[b]
PC (16:0/22:3)	1.77E+07±3.02E+07[a]	7.86E+07±3.05E+07[b]	4.09E+07±3.89E+07[ab]
PC (16:0e/2:0)	8.45E+08±4.27E+08[a]	8.35E+08±3.59E+08[a]	5.82E+08±2.24E+08[a]
PC (16:0e/20:3)	1.37E+08±7.79E+07[a]	3.52E+07±2.64E+07[b]	2.95E+07±2.66E+07[b]
PC (18:0/20:5)	1.81E+09±4.78E+08[a]	1.35E+09±2.76E+08[ab]	1.21E+09±3.75E+08[b]
PC (18:3e/2:0)	1.57E+08±6.91E+07[a]	6.56E+07±3.82E+07[b]	1.24E+08±4.14E+07[a]
PC (18:5e/16:0)	5.05E+08±2.23E+08[a]	1.08E+08±5.24E+07[b]	1.13E+08±1.15E+08[b]
PC (18:5e/16:3)	1.43E+08±5.24E+07[a]	3.79E+08±1.43E+08[b]	1.17E+08±5.10E+07[a]
PC (22:1/18:5)	7.90E+07±1.00E+08[a]	2.06E+08±1.54E+08[b]	1.94E+08±9.19E+07[b]
PE (18:1/18:1)	1.05E+08±8.34E+07[a]	2.09E+08±1.15E+08[a]	1.22E+08±7.70E+07[a]
PE (2:0/20:0)	8.12E+07±3.24E+07[a]	1.70E+07±9.70E+06[b]	2.43E+07±2.27E+07[b]
PE (3:0/16:2)	1.39E+08±4.66E+07[a]	3.50E+07±1.79E+07[b]	3.90E+07±3.65E+07[b]
PG (2:0/4:0)	1.65E+07±4.13E+07[a]	1.33E+08±4.76E+07[b]	6.42E+07±3.62E+07[ab]
PG (2:0/5:0)	4.10E+07±5.44E+07[a]	4.44E+07±4.01E+07[a]	1.55E+08±1.44E+08[b]
PI (2:0/13:1)	1.14E+08±7.51E+07[a]	1.24E+06±4.47E+05[b]	2.33E+07±7.08E+07[b]
PI (2:0/17:2)	4.06E+07±6.16E+07[a]	8.91E+07±3.78E+07[ab]	1.28E+08±5.95E+07[b]
PI (21:0/17:1)	1.07E+08±5.68E+07[a]	2.39E+06±1.07E+06[b]	1.22E+07±3.03E+07[b]
PI (4:0/16:2)	4.55E+07±6.24E+07[a]	9.73E+07±3.08E+07[b]	1.60E+08±8.46E+07[b]
PI (4:0/19:2)	2.36E+07±1.82E+07[a]	8.14E+07±7.29E+07[b]	1.36E+08±9.77E+07[b]
PI (6:0/9:0)	1.01E+08±6.64E+07[a]	1.77E+06±4.08E+05[b]	2.15E+06±2.53E+06[b]
